# A Narrative Review of the Clinical Applications of Echocardiography in Right Heart Failure

**DOI:** 10.3390/jcm14155505

**Published:** 2025-08-05

**Authors:** North J. Noelck, Heather A. Perry, Phyllis L. Talley, D. Elizabeth Le

**Affiliations:** 1 Division of Cardiology, Portland Veterans Administration Health Care System, Portland, OR 97239, USA; north.noelck2@va.gov (N.J.N.);; 2 Knight Cardiovascular Institute, Division of Cardiovascular Medicine, Oregon Health & Science University, Portland, OR 97239, USA

**Keywords:** right atrium, right ventricle, right heart failure, RV function assessment, echocardiography, 3D echocardiography, quantification

## Abstract

**Background/Objectives:** Historically, echocardiographic imaging of the right heart has been challenging because its abnormal geometry is not conducive to reproducible anatomical and functional assessment. With the development of advanced echocardiographic techniques, it is now possible to complete an integrated assessment of the right heart that has fewer assumptions, resulting in increased accuracy and precision. Echocardiography continues to be the first-line imaging modality for diagnostic analysis and the management of acute and chronic right heart failure because of its portability, versatility, and affordability compared to cardiac computed tomography, magnetic resonance imaging, nuclear scintigraphy, and positron emission tomography. Virtually all echocardiographic parameters have been well-validated and have demonstrated prognostic significance. The goal of this narrative review of the echocardiographic parameters of the right heart chambers and hemodynamic alterations associated with right ventricular dysfunction is to present information that must be acquired during each examination to deliver a comprehensive assessment of the right heart and to discuss their clinical significance in right heart failure. **Methods:** Using a literature search in the PubMed database from 1985 to 2025 and the Cochrane database, which included but was not limited to terminology that are descriptive of right heart anatomy and function, disease states involving acute and chronic right heart failure and pulmonary hypertension, and the application of conventional and advanced echocardiographic modalities that strive to elucidate the pathophysiology of right heart failure, we reviewed randomized control trials, observational retrospective and prospective cohort studies, societal guidelines, and systematic review articles. **Conclusions:** In addition to the conventional 2-dimensional echocardiography and color, spectral, and tissue Doppler measurements, a contemporary echocardiographic assessment of a patient with suspected or proven right heart failure must include 3-dimensional echocardiographic-derived measurements, speckle-tracking echocardiography strain analysis, and hemodynamics parameters to not only characterize the right heart anatomy but to also determine the underlying pathophysiology of right heart failure. Complete and point-of-care echocardiography is available in virtually all clinical settings for routine care, but this imaging tool is particularly indispensable in the emergency department, intensive care units, and operating room, where it can provide an immediate assessment of right ventricular function and associated hemodynamic changes to assist with real-time management decisions.

## 1. Introduction

**Figure 1:**
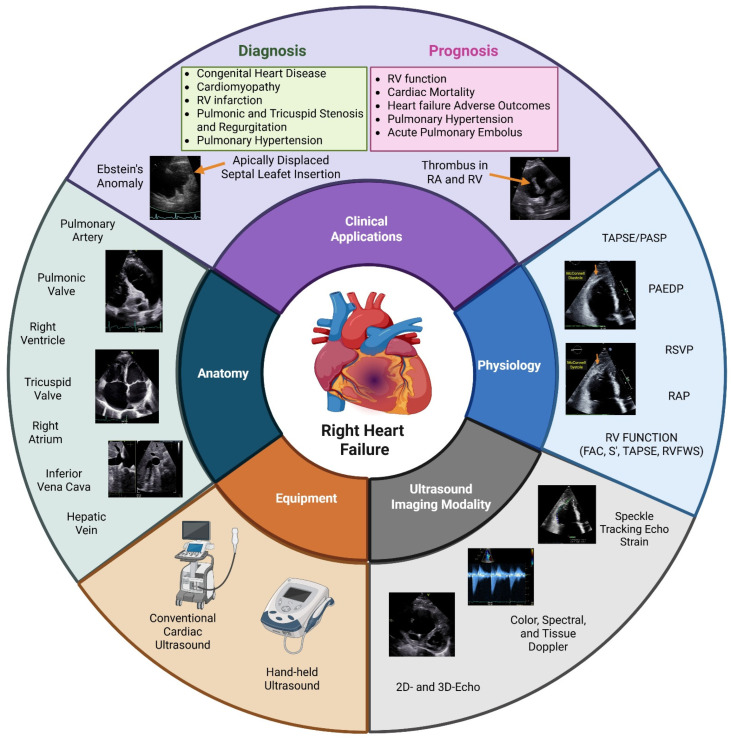
Multiple echocardiography imaging modalities can determine the anatomy and function of the right heart using both conventional and hand-held cardiac ultrasound. The echocardiographic measurements are both diagnostic and prognostic. RV—right ventricle; TAPSE—tricuspid annular plane systolic excursion; PASP—pulmonary artery systolic pressure, PAEDP—pulmonary artery end-diastolic pressure; RVSP—right ventricular systolic pressure; RAP—right atrial pressure; FAC—fractional area change; S′—tricuspid lateral annular systolic velocity; RVFWS—right ventricular free wall longitudinal strain; 2D—two-dimensional; 3D—three-dimensional. Created in https://BioRender.com.

The right ventricle (RV) has historically been neglected, in part due to its wedge shape, rendering it challenging to image with cardiac ultrasound, and in part due to an under-appreciation of its important functionality despite the interdependence with the left ventricle (LV). Innovation in the field of echocardiography over the past two decades has enabled providers to appreciate the intricate composition of this chamber, to develop two-dimensional and Doppler measurements that reflect the RV’s adaptation to preload and afterload, and to determine the role of these echocardiographic metrics in the clinical management of acute and chronic right heart failure. The primary objective of this narrative review is to present contemporary echocardiographic measurements of the right heart chambers and hemodynamic alterations associated with RV dysfunction that are required to provide a comprehensive assessment of the right heart and discuss their impact on the clinical care of right heart failure patients (Figure 1).

## 2. Methods

We performed a literature search in the PubMed database from 1985 to 2025 and the Cochrane database, which included but was not limited to the following terminology, phrases, or combination of phrases: right ventricular anatomy, right heart anatomy, right ventricular development, right ventricle, right ventricular failure, acute and chronic right heart failure, right ventricle function, right heart function, right ventricular–pulmonary arterial coupling, pulmonic valve disease, tricuspid valve disease, cardiomyopathy, chemotherapy, pulmonary embolus, congenital heart defects, arrhythmogenic right ventricular cardiomyopathy, left atrial function, echocardiography, noninvasive imaging of the right heart, stress echocardiography, global and regional strain analysis, speckle-tracking echocardiography, cardiac magnetic resonance imaging of the right ventricle and right heart, artificial intelligence, machine learning, and automation. Inclusion criteria included studies published in the English language between 1984 and 2025, randomized controlled trials, observational retrospective and prospective cohort studies involving participants aged ≥ 18 years recruited from any racial/ethnic population groups, the most recent societal guidelines, and systematic review articles. We excluded case reports, editorials, and abstracts. The full-length publications were reviewed, and their findings were summarized to provide a concise presentation of the clinical utility and applications of echocardiographic parameters in the diagnosis and contemporary management of right heart failure in the context of societal guidelines recommendations. Where possible, we compared echo-derived parameters with magnetic resonance imaging-derived measurements.

Since this work is not a systematic review, the limitations of this narrative review include selection bias, retrieval bias, language bias, and publication bias.

This study is a clinical narrative literature review and does not contain a collection of primary human data; thus, institutional review board approval, informed consent, and dataset availability locations are not applicable.

## 3. Right Ventricular Anatomy

The embryologic development of the RV is distinct from the LV [1]. The RV develops from the secondary heart field while the LV develops from the primary field, and myocytes from each ventricle differentiate under dissimilar genetic programming. The RV myocardium has only two layers of fibers, circumferential and deep longitudinal, with wall thickness 2–3 mm, coarse trabeculations, and several papillary muscles, whereas the LV myocardium has three layers, circumferential, oblique, and longitudinal, with wall thickness 7–11 mm, fine trabeculations, and commonly two papillary muscles. The RV has a crescentic shape and is supplied by one coronary artery, whereas the LV has an ellipsoidal shape and is supplied by two or three coronary arteries [2]. RV contraction relies on longitudinal shortening and is very dependent on loading conditions [3]. Despite these morphologic and functional differences, the adult RV, a high-volume and low-pressure pump, generates a similar cardiac output as the LV at only one-sixth the energy expenditure [4].

## 4. Echocardiographic Assessment of the Right Heart

Echocardiographic assessment of the right heart begins with analyzing the size of the right-sided chambers, including the right atrium (RA), the RV, the inferior vena cava (IVC), and, if possible, the main pulmonary artery (MPA). RV wall thickness (RVWT) and RV systolic function provide additional insight. Furthermore, the estimation of right atrial pressure (RAP) is integrated with the measured RV size and thickness and RV systolic function to suggest further diagnostic possibilities or treatment plans. Standard imaging acquisition must include the parasternal long (PLAX) and short axes (PSAX), the apical 4-chamber (A4C), the dedicated RV, and the subcostal views.

### 4.1. Right Atrial and Ventricular Size

RA size is determined through an optimized A4C view, which maximizes the chamber size in ventricular systole (atrial diastole). The RA area (<19 cm^2^) or an indexed RA calculated volume (area–length < 33 mL/m^2^ or method of disks < 30 mL/m^2^) may be used to determine RA dilatation [5]. A dilated RA may suggest elevated right-sided pressures and volume overload or arrhythmias.

**Figure 2:**
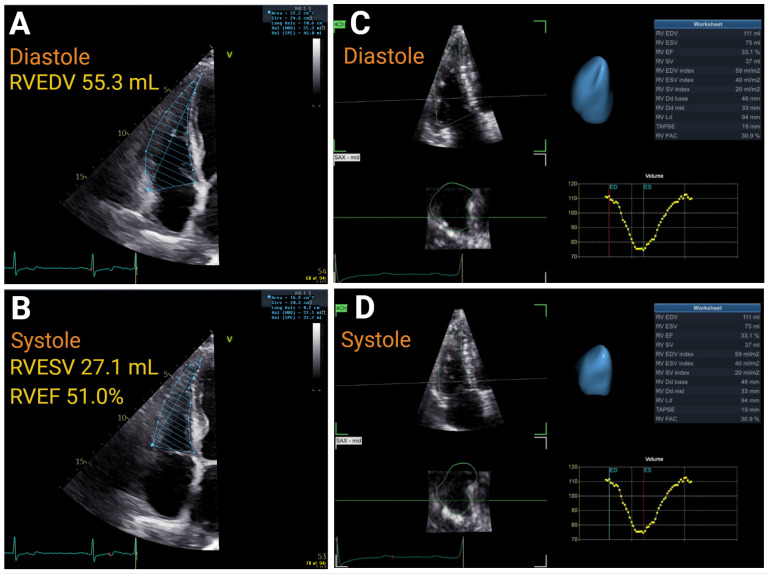
The 2D and 3D RV volumetric assessment of RV function: 2DE diastole Panel (**A**) and 2DE systole Panel (**B**); 3DE diastole Panel (**C**) and 3DE systole Panel (**D**). RVEDV—right ventricular end-diastolic volume; RVESV—right ventricular end-systolic volume; RVEF-right ventricular ejection fraction. Created in htpps://BioRender.com.

RV size may be assessed with two-dimensional echocardiography (2DE) via linear or area measurements and three-dimensional echocardiography (3DE) volumes (Figure 2). Given the atypical geometry of the RV, it is recommended to assess size from multiple windows and synthesize this data. Normal values are reported for linear measurements, areas, and volumes (Table 1) [5].

Cardiac magnetic resonance imaging (cMRI) is considered the exemplar technique for the assessment of the RV in clinical practice; however, the echocardiogram remains the first-line test given portability, cost, and availability advantages. Nonetheless, the use of echocardiographic measurements which correlate well with abnormal RV on cMRI may be valuable (Table 1). The best agreement between ultrasound and cMRI may be found with the basal RV diameter in A4C (r = 0.70), proximal RVOT diameter (r = 0.68) in PLAX [6], and RV end-diastolic area (r = 0.76) [7]. Prospective analysis in two small studies (*n* < 50) found that the use of 3DE systematically underestimates RV sizes by an average of 26–88 mL depending on technique; therefore, a direct comparison of volumes between these two techniques is less clinically useful [8,9].

The MPA diameter may be measured in the PSAX in the pulmonary artery (PA) bifurcation view and should be <2.5 cm in diameter and/or the ratio of the MPA to ascending aorta diameter should be <1.

IVC measurements are obtained in dedicated subcostal images and, ideally, are performed with a concurrent orthogonal axis. The IVC diameter (normal <2.2 cm) is obtained during end-expiration and is measured 0.5 to 3.0 cm near the RA ostium [5]. A correct identification of the IVC can be confirmed with the visualization of hepatic veins flowing into the IVC and termination of the vessel in the RA.

### 4.2. Right Ventricular Thickness

The measurement of RVWT (normal < 0.5 cm) should be obtained from the anteromedial aspect of the RV free wall using a subcostal view. If this window is sub-optimal, the RVWT may be obtained from the PLAX. Standardized reporting RVWT is only expected if abnormal and, like abnormalities in RV size, it has been correlated with an adverse prognosis. Right ventricular hypertrophy (RVH) may be associated with remodeling in the setting of pulmonary hypertension (PH); however, approximately 1 in 5 patients with RVH have normal pulmonary artery systolic pressure (PASP). In these situations, consideration of alternative diagnoses including hypertrophic cardiomyopathy (HCM) and infiltrative cardiomyopathies is warranted. There is a fair correlation (r = 0.71) between RVH demonstrated in the subcostal window on echocardiogram with cMRI in a retrospective analysis of 200 subjects [10].

### 4.3. Right Atrial Function

An emerging technique that can be utilized to assess right atrial function is the measurement of right atrial strain using speckle-tracking echocardiography (STE). Similar to the left atrium, right atrial strain has three phases: reservoir function, conduit function, and contractile function. While several studies have demonstrated the prognostic value of right atrial strain in small cohorts with varied cardiac conditions, a large systemic review and meta-analysis of 21 studies involving 4111 subjects reported that the normal range of right atrial strain varied widely, which may reduce its utility in clinical practice [11]. Nevertheless, right atrial strain has been shown to correlate with poor outcomes in a prospective cohort of 104 patients with idiopathic pulmonary hypertension [12], to be associated with right heart failure and overload independent of RA size and pressure in a randomized study of 65 patients with advanced pulmonary hypertension [13], to independently predict mortality and hospitalization and in a prospective study of 63 patients with pulmonary hypertension and heart failure [14], and to independently predict long-term outcome and the extent of right heart failure over 19.3 ± 10.9 months in a prospective study of 53 patients with connective tissue disease-associated pulmonary arterial hypertension [15]. In addition, it has predicted atrial fibrillation occurrence in 73 patients with secundum atrial septal defect treated with occluder insertion within 6 months [16] and atrial fibrillation recurrence in 56 patients who were treated with catheter ablation within 7 ± 3 months [17]. Right atrial strain analysis is straightforward and can be performed expeditiously, but reliable and reproducible data are greatly dependent on optimal image quality. Further validation studies in subpopulations and disease states are warranted before right atrial strain can become a practical clinical tool.

### 4.4. Right Ventricular Function

**Figure 3:**
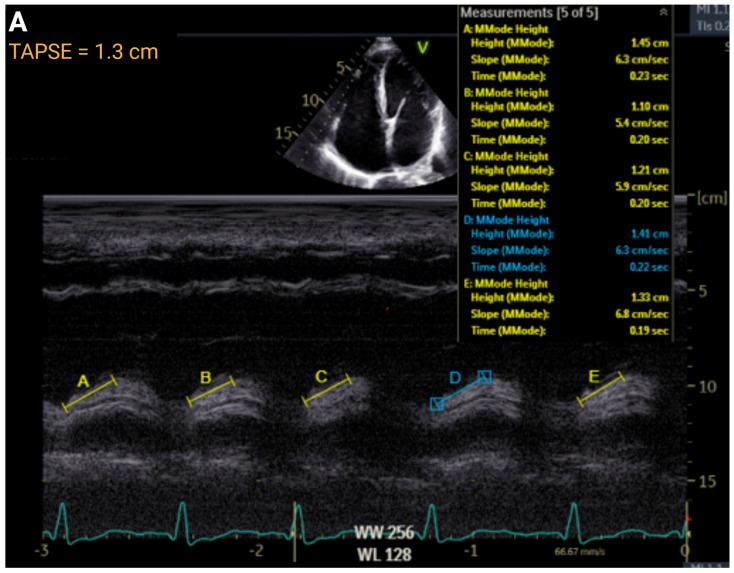

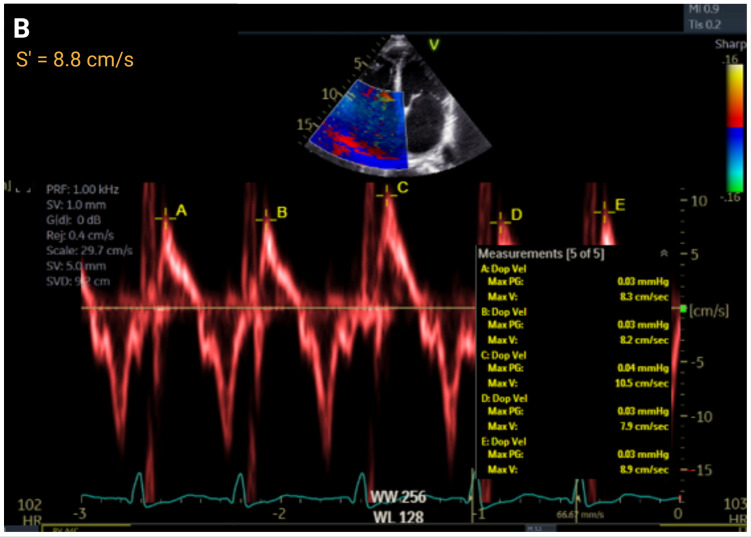

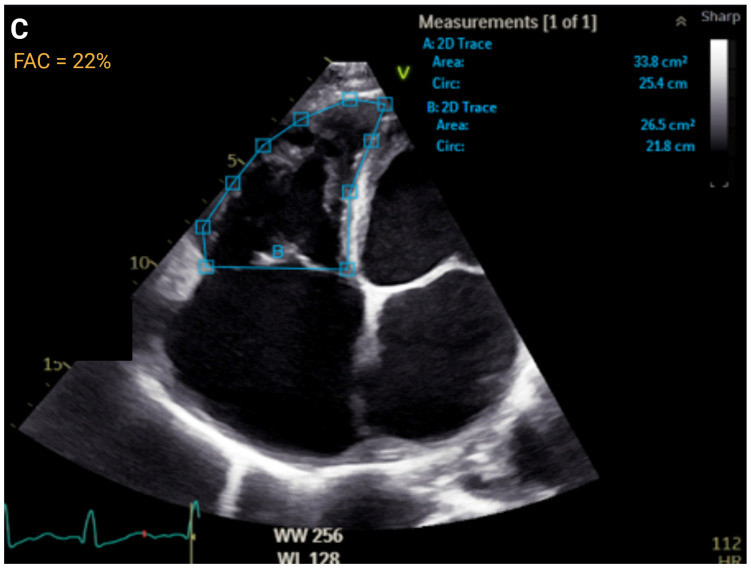

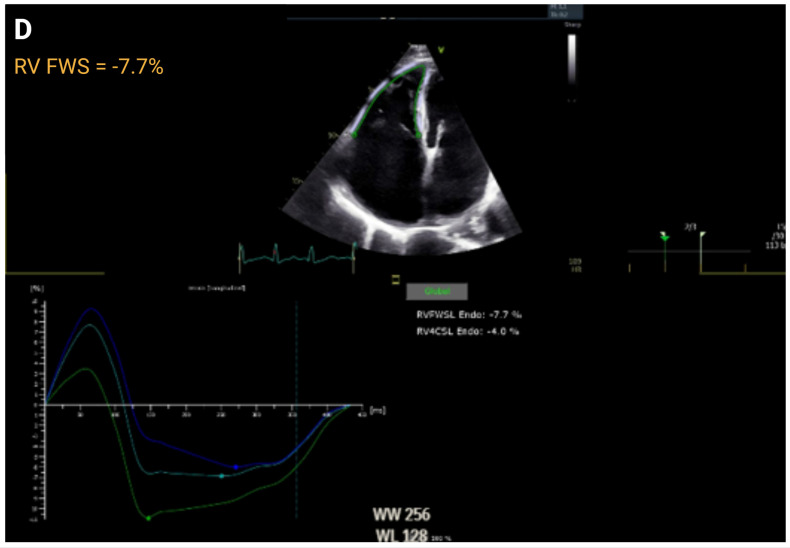
Representative echocardiographic methods of right ventricular function assessment. Panel (**A**): Tricuspid annular plane systolic excursion (TAPSE). Panel (**B**): Tissue Doppler imaging of the tricuspid lateral annular systolic velocity (S′). Panel (**C**): right fractional area change (FAC). Panel (**D**): Right ventricular free wall longitudinal strain (RVFWS). Created in https://BioRender.com.

The importance of RV function has taken on increased prominence in the PH and heart failure (HF) community over the past several decades. In that time, a deeper understanding of RV pathophysiology has occurred in tandem with how echocardiographic tools may provide insight regarding clinical status. Common echocardiographic parameters to assess RV systolic function on echocardiogram are tricuspid annular plane systolic excursion (TAPSE), RV fractional area change (FAC), and tissue Doppler imaging tricuspid lateral annular systolic wave velocity (S′) (Figure 3). Each of these parameters has been demonstrated to have prognostic value in various clinical contexts independent of clinical factors, natriuretic peptides, or other echocardiographic variables. Interestingly, these values may be discordant in approximately 1/3 of echocardiograms [18].

TAPSE (normal > 1.7 cm) was the earliest echocardiographic method for assessing RV dysfunction. It was validated against radionuclide imaging in 1984 and remains a clinically relevant assessment of the longitudinal contraction of the RV [19]. Over four decades, the utility of TAPSE has been demonstrated in a variety of clinical contexts, including HF, PH, emphysema, diabetes, hypertension, aortic stenosis, arrhythmogenic RV cardiomyopathy (ARVC), atrial fibrillation, and ischemic heart disease [20]. In an observational prospective study of 56 patients, TAPSE had a fair correlation with MRI as a predictor of RV dysfunction [7]. S′ (normal > 9.5 cm/s) and TAPSE provide similar clinical information about the longitudinal function of the RV and generally provide similar clinical information [18].

Compared with TAPSE and S′, FAC (normal > 35%) is a 2DE attempt to integrate both the longitudinal and radial components of RV function. The development of RV dysfunction as assessed by FAC in patients with chronic HF with preserved ejection fraction (HFpEF) has been demonstrated to be associated with adverse outcomes, including increased mortality (HR 1.82, 95% CI 1.01–3.19; *p* = 0.04) in a prospective study of 271 patients [21]. Similarly, in a prospective study cohort of 480 hospitalized subjects, a reduction in FAC following an acute HF exacerbation may portend increased all-cause mortality (HR 2.33, 95% CI 1.58–3.43, *p* < 0.01) [22]. FAC outperforms linear measurements in terms of agreement with cardiac MRI in assessing RV dysfunction [7].

The use of RV strain imaging in echocardiography is an emerging technique that is feasible and reproducible when image quality is sufficient. RV STE may be obtained in 2DE and 3DE. In 2DE, STE may be performed as RV longitudinal free wall strain (RVFWS) and RV global longitudinal strain (RVGLS). RVFWS (normal is more negative than −20%) incorporates three segments of the RV free wall obtained in an A4C window divided into apical, mid, and basal segments. RV GLS (normal is more negative than −20%) includes the three segments of RV FWS and three additional segments from the septum, as obtained in A4C. Clinically, RVFWS is usually the preferred method of assessment as it avoids confounding from LV longitudinal strain, particularly in patients with LV systolic dysfunction. In a prospective study of 288 subjects, like TAPSE, S′, and FAC, RVFSW may provide incremental prognostic value to other clinical markers particularly in patients with heart failure [23].

Interestingly, despite a systematic underestimation of the RV size by 3DE compared to CMR, the calculated RV ejection fraction (EF) from the two techniques is comparable. On average, 3DE was within 2–3% of CMR-calculated EF, though the level of agreement was between +/−5 and +/−16%, depending on the technique. Three-dimensional echocardiography may be feasible and reproducible in patients with excellent RV endocardial definition on echocardiogram [8,9].

A novel noninvasive technique to assess myocardial performance that is load-independent is myocardial work (MW). This technique incorporates noninvasive systolic blood pressure with global strain data to derive the global work index, global constructive work, global wasted work, and global work efficiency [24]. These parameters are calculated from vendor-specific algorithms after the input of the peak arterial pressure. Compared to the left ventricle, the MW of the right ventricle has not been as extensively validated. However, RV global work efficiency has been shown to be associated with mortality in a small cohort of patients who underwent left ventricular assist device implantation [25], and RV global constructive work and RV global work index were associated with all-cause mortality in a cohort of 51 patients with pulmonary hypertension [26]. While promising, this technique, similar to GLS analysis, is not reliable in patients with poor image quality and irregular rhythm. Furthermore, RV-specific software needs to be developed and validated.

Other methods for assessing right ventricular function such as the instantaneous rise in pressure over time (dP/dT, normal > 400 mmHg/s) and the myocardial performance index (MPI, normal < 0.55 TDI method) have generally fallen out of use in favor of the previously discussed methods.

RV diastolic dysfunction is generally alluded to in the echocardiographic report by various measures, including tricuspid valve inflow Doppler E/A ratio and deceleration time, E/e′, and hepatic vein (HV) flow patterns. Practically, this information is integrated in the calculation of right-sided pressures through their impact on the estimation of RAP. Elevated right-sided filling pressure estimates may precede RV dilation and RVH, making recognition important in the clinical management of patients with HF and PH.

#### Hemodynamic Assessment

The echocardiogram can provide a reasonable estimation of mean RAP, RV systolic pressure (RVSP), mean pulmonary artery pressure (mPAP), and pulmonary artery end-diastolic pressure (PAEDP). The inclusion of noninvasive estimates of right-sided filling pressure may be clinically useful and augment the understanding of anatomic and functional findings on the echocardiogram.

An estimation of RAP relies on the integration of multiple measurements. Current guidelines begin with assessing the size (normal ≤ 2.1 cm) and collapsibility index (≥50%) of the IVC. An IVC diameter > 2.0 cm demonstrates a good correlation with elevated RAP (>10 mmHg, r = 0.76–0.91) in patients who were assessed breathing comfortably in the supine position [27]. It is important to ensure that the IVC size represents the true diameter of the vessel, and thus a concomitant orthogonal view of the IVC is highly recommended. In a small prospective cohort of 35 subjects, a dilated IVC does not correlate (r = 0.4) with elevated RAP in patients who are mechanically ventilated; however, a normal IVC diameter may be accurate in identifying patients with RAP < 10 mmHg [28]. Studies attempting to correlate IVC size to body surface area (BSA) have produced mixed results and, as such, indexing for BSA is not routinely recommended [28].

IVC collapsibility (normal > 50% with sniff and quiet breathing) is determined based on the change in IVC diameter from end-expiration to end-inspiration. An IVC collapsibility < 50% with sniff or, in patients unable to sniff < 20% with quiet breathing, correlates with elevated RAP pressures on invasive testing. The use of M-mode imaging during the breathing cycle may assist with collapsibility variation determination. Individual studies may conclude that the use of IVC diameter is favored over IVC collapsibility and vice versa to predict RAP; additionally, there are some studies that favor the combination of both. Small sample size, variation in study population, acuity of disease state, techniques, and reference cut-points all play a role in determining study conclusions (Table 2).

**Figure 4:**
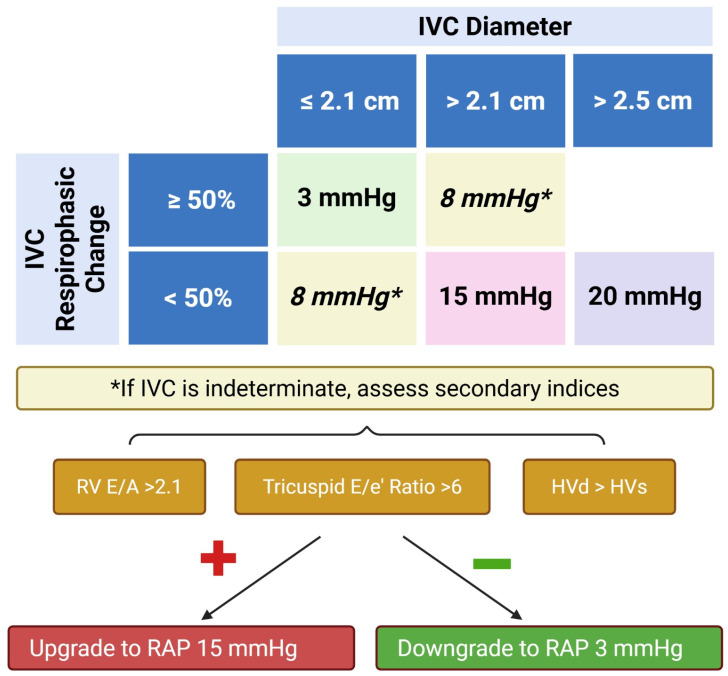
Algorithmic determination of right atrial pressure according to echocardiographic parameters, adapted from Figure 3 with permission from Elsevier [5]. IVC—inferior vena cava; RV—right ventricular; HVd—hepatic vein diastole; HVs—hepatic vein systole; RAP—right atrial pressure. Created in https://BioRender.com.

In general, if both the IVC size and collapsibility are abnormal, then elevated RAP may be concluded (15 mmHg). Similarly, if both values are normal, then normal RAP may be assumed (3 mmHg) [5]. When size and collapsibility are discordant, guidelines suggest integrating additional data points prior to using an indeterminate (8 mmHg) estimate of RAP. In this situation, a review of RA size, interatrial septal movement throughout the cardiac cycle, and consideration of RV diastolic function (i.e., tricuspid inflow deceleration time (DT), tricuspid Doppler E/A ratio, tricuspid E/e′ ratio, and HV Doppler flow) are indicated (Figure 4). In addition, the American Society of Echocardiography (ASE) Guidelines suggest that, if the end-expiration IVC is >2.5 cm and lacks respirophasic variation, then 20 mmHg can be considered [5]. Finally, it should be noted that, while the ASE Guidelines recommend the use of absolute RAP values, the British Society of Echocardiography endorses using a range of pressures: normal (0–5 mmHg), intermediate (5–10 mmHg), and high (>15 mmHg) [29].

**Table 2:** Diagnostic probability and correlation of echocardiographic parameters compared to invasive hemodynamic assessment.

	Cut-Point	Invasive Pressure, mmHg	Sensitivity	Specificity	NPV	PPV	ROC Curve	Correlation Coefficient	N
**IVC Diameter, cm**									
Brennan JM et al. 2007 [30]	>2.0	RAP ≥ 10 mmHg	0.73	0.85	0.62	0.9	AUC = 0.76	r = 0.5	102
Egbe AC et al. 2002 [31]	>2.0	RAP > 10 mmHg	0.63	0.89	0.57	0.74	AUC = 0.67	r = 0.56	918
Moreno FL et al. 1984 [32]	>2.3	RAP > 7 mmHg	0.4	0.97	0.58	0.93	NR	NR	65
Prekker ME et al. 2013 [33]	<2.0	CVP < 10 mmHg	0.85	0.81	0.87	0.78	AUC = 0.91	r = 0.76	65
Taniguchi T et al. 2015 [34]	>2.0	RAP ≥ 10 mmHg	NR	NR	NR	NR	AUC = 0.83	r = 0.67	90
Utsunomiya H et al. 2009 [35]	>2.3	RAP > 10 mmHg	0.87	0.83	NR	NR	AUC = 0.85	NR	50
**Respirophasic Variation—IVC Collapsibility % (sniff)**									
Brennan JM et al. 2007 [30]	<40	RAP ≥ 10 mmHg	0.73	0.84	0.62	0.9	AUC = 0.91	NR	102
Egbe AC et al. 2002 [31]	<50	RAP > 10 mmHg	85	72	80	78	AUC = 0.76	r = 0.72	918
Kircher BJ et al. 1990 [36]	<50	RAP ≥ 10 mmHg	87	82	NR	NR	NR	r = 0.75	83
Taniguichi T et al. 2015 [34]	<50	RAP ≥ 10 mmHg	NR	NR	NR	NR	NR	NR	90
**Respirophasic Variation—IVC Collapsibility % (passive)**									
Brennan MJ et al. 2007 [30]	<20	RAP > 10 mmHg	0.73	0.82	0.57	0.9	AUC = 0.93	NR	102
Moreno FL et al. 1985 [32]	<40	RAP > 7 mmHg	0.91	0.9	0.9	0.91	NR	NR	65
Nagueh SF et al. 1996 [28]	<50	RAP > 8 mmHg	0.72	0.75	NR	NR	NR	NR	85
Prekker ME et al. 2013 [33]	>50	CVP < 10 mmHg	0.47	0.77	0.75	0.5	AUC = 0.66	NR	65
Taniguichi T et al. 2015 [34]	<25	RAP ≥ 10 mmHg	NR	NR	NR	NR	AUC = 0.79	NR	90
**IVC Diameter + Respirophasic Variation**									
Arbo JE et al. 2013 [37]	≥1.9 cm and >50%	CVP ≥ 10 mmHg	0.46	0.9	0.75	0.72	NR	NR	30
Egbe AC et al. 2002 [31]	≥2.1 cm and <50% (sniff)	RAP > 10 mmHg	65	99	55	89	AUC = 0.76	r = 0.681	918
**Doppler—E/A Ratio**									
Nagueh SF et al. 1996 [28]	≥1.1	RAP > 8 mmHg	NR	NR	NR	NR	NR	r = 0.66	85
**Tissue Doppler Imaging—E/e**′									
Arbo JE et al. 2013 [37]	>4.6	CVP ≥ 10 mmHg	0.85	0.9	0.85	0.9	NR	NR	30
Nagueh SF et al. 1996 [28]	>6	RAP > 8 mmHg	0.79	0.73	NR	NR	NR	NR	85
Sade LE et al. 2007 [38]	≥4	RAP > 10	0.88	0.85	NR	NR	AUC = 0.93	r = 0.83	53
Said K et al. 2012 [39]	≥4.5	RAP > 10 mmHg	0.89	1	NR	NR	AUC = 0.95	r = 0.84	50
Sundereswaran L et al. 1998 [40]	>8	RAP ≥ 10 mmHg	0.78	0.85	NR	NR	NR	r = 0.63	50
Utsunomiya H et al. 2009 [35]	>7.3	RAP > 10	0.87	0.97	NR	NR	AUC = 0.92	r = 0.8	50
**Hepatic Vein Doppler Flow Pattern—Hepatic Vein Systolic Filling Fraction, %**									
Nagueh SF et al. 1996 [28]	Vs/(Vs + Vd) < 55	>8	86	92	NR	NR	NR	r = 0.86–0.92	85

NPV—negative predictive value; PPV—positive predictive value; ROC—receiver operator characteristic; N—number; RAP—right atrial pressure; CVP—central venous pressure; NR—not reported; AUC—area under curve.

Increased RA size or evidence of the interatrial septum protruding into the left atrium are both suggestive of elevated RAP. Upgrading from indeterminate (8 mmHg) to elevated (15 mmHg) RAP is also suggested when any of the following are present: a restrictive RV diastolic filling pattern defined as E/A > 2.1, DT < 120 msec, E/e′ > 6, or a diastolic predominance in HV flow.

After the IVC diameter and collapsibility, the E/e′ ratio is the best-studied metric and demonstrates a moderate to strong correlation (r = 0.77–0.84) with an invasive assessment of RAP (Table 2). In 42 mechanically ventilated patients, a group in which RAP cannot be reliably estimated with IVC diameter or collapsibility, the E/e′ has a good correlation (r = 0.77) with RAP [38]. Some evidence suggests that the serial measurement of E/e′ may be used to track changes in RAP over time; in a small subset of patients in two studies, an E/e′ increase >2 was associated with an increase of >5 mmHg in RAP [38,40]. Unfortunately, there are several populations where there is no correlation between E/e′ and RAP, notably in patients with recent cardiac surgery (r = 0.41) [38]. Additionally, many studies of E/e′ and RAP specifically excluded patients with significant tricuspid regurgitation (TR), atrial fibrillation, and ventricular pacing due to confounding.

Once an estimation of RAP is established, the determination of RVSP, MPAP, and PAEDP may proceed. RVSP is most commonly calculated through the simplified Bernoulli equation using the peak tricuspid regurgitant jet and adding this value to the estimated RAP: RVSP = estimated RAP + 4(peak TR velocity)^2^. This method relies on an adequate tricuspid regurgitant jet. An inadequate continuous wave signal is the most common cause of both the overestimation and underestimation of pressure [41]. Other causes of an overestimation of RVSP include not selecting the modal frequency, “over-gaining” the TR jet signal, arrhythmia, and severe TR. Underestimation may be caused by an unfavorable angle (i.e., >20 degrees between ultrasound beam and direction of blood velocity). Care should be taken to exclude cases where the TR signal is faint and incomplete. To overcome this deficit, ultrasound enhancing agents have been demonstrated to significantly improve the TR spectral Doppler signal quality [42]. Despite these challenges, the clinically reported RVSP on echocardiogram has a strong correlation (0.84) with right heart catheterization (RHC) data and is reliably within 20% of the invasive RVSP [20].

Estimates of PAEDP can also be performed using the simplified Bernoulli equation and the pulmonic valve regurgitant jet. PAEDP is calculated by multiplying the square of the end-diastolic velocity and adding the estimated RAP: PAEDP = RAP + 4(end-diastolic PR velocity)^2^ [5]. The MPAP may be estimated using a number of methods, including MPAP = 1/3 × PASP + 2/3 × PAEDP, the use of RV outflow tract (RVOT) acceleration time (AT; MPAP = 79 − (0.45 × AT)) in msec, peak pulmonic valve regurgitation (PR; MPAP = 4 × early PR velocity)^2^ + RAP)) in m/s, or estimated RVSP (MPAP = 0.6 × RVSP + 2.1 mmHg).

Estimates of RVSP are highlighted in recent guidelines for PH screening and monitoring. The recently revised threshold of MPAP > 20 mmHg correlates to an RVSP of approximately 30 mmHg [43]. ASE guidelines make the inference that, if the TR jet is ≥2.9 m/s or if the peak TR jet is ≥2.8 m/s with supportive echocardiographic features of PH or with known clinical risk factors for PH, then PH should be suspected. Echocardiographic features consistent with PH include the dilation of the RA, RV, MPA, or IVC, RVH, a short RVOT AT (<105 msec), an abnormal RV eccentricity index (EI), abnormal RV function, or the presence of a pericardial effusion. The EI is calculated by dividing the RV diameter parallel to the interventricular septum by the RV diameter perpendicular to the septum; EI < 1 is normal, and EI > 1 supports RV overload.

While it is possible to calculate pulmonary vascular resistance (PVR) and pulmonary capillary wedge pressure (PCWP) noninvasively, these techniques are rarely applied. The calculation of PVR relies on indirect measurements, and small errors in several measurements can significantly impact accuracy. Similarly, the derived calculation for PCWP has limited clinical utility in wide swaths of patients with cardiovascular conditions, including arrhythmias, valvular heart disease, and pacing.

### 4.5. Right Ventricular–Pulmonary Arterial Coupling and Uncoupling

RV–PA coupling is a key determinant in clinical outcomes in PH and HF. The gold standard for the assessment of RV–PA coupling is the invasive derivation of RV pressure volume loops and the calculation of the end-systolic elastance (Ees) to pulmonary arterial elastance (Ea). Using this ratio of contractile performance relative to RV afterload is an attempt to quantify RV efficiency. A normal ratio indicates efficient energy transfer from the RV into the pulmonary circulation. A reduction in this ratio indicates a mismatch between RV contractility and PA afterload. If there is an acute increase in pulmonary pressure, the RV will initially compensate by increasing contractility. When elevated pulmonary pressures persist or increase, RV contractile reserve is eventually exhausted, leading to RV–PA uncoupling. Over time, remodeling will occur along a continuum, starting with RVH, followed by minimal RV dilation and preserved RV function. Remodeling will eventually become maladaptive with significant RV dysfunction and chamber dilation. In later stages, the RV may define the cardiac apex in A4C views.

Measures of RV contractile performance (TAPSE, S′, and RV FWS) and estimates of hemodynamic load (RVSP) may serve as a surrogate for invasively derived Ees/Ea ratio. Abnormalities in TAPSE/PASP or RVFWS/PASP have been associated with poor functional status and prognosis in a wide variety of clinical conditions, including HFpEF, transcatheter aortic valve replacements, transcatheter mitral valve interventions, transcatheter tricuspid valve interventions, systemic lupus erythematosus-associated PH, acute pulmonary embolism (PE), chronic thromboembolic PH, chronic PH, and COVID-19-associated acute respiratory distress syndrome [44]. A normal TAPSE/PASP ratio is ≥0.55 mm/mmHg, assuming the absence of RVOT obstruction or pulmonic valve stenosis, and can be readily calculated based on data already obtained during most echocardiography studies. A prospective study of 293 patients demonstrated that clinical prognosis declines as the TAPSE decreases and PASP increases, with a cut-point for substantial morbidity and mortality occurring between 0.3 and 0.4 mm/mmHg [45]. RVFWS/PASP is an emerging tool to assess early RV–PA uncoupling in disease states where a 2DE assessment of RV function is normal or only mildly impacted such as cardiac amyloid and HCM.

## 5. Exercise Hemodynamics and Right Ventricular Functional Reserve

Exercise provides insight into cardiopulmonary function in health and disease. A hemodynamic stress test may uncover PH in patients with symptoms of dyspnea or exercise intolerance and may assist in prognostication in patients with known PH. Echocardiographic measurements are obtained at rest and during semi-recumbent bicycle exercise. Standard metrics include an RV functional measure (TAPSE, S′, FAC; RVFWS if technically feasible), RVSP (RAP is estimated based on resting echo parameters), LV diastolic parameters (if left heart disease is suspected), and LVOT VTI to calculate a noninvasive estimate of cardiac output (CO based on CO = SV × HR, SV = LVOT VTI × LVOTd).

Exercise augments stroke volume (SV) in normal subjects, which is accomplished by a modest increase in RV size (~20% by standard 2DE measurements) and RV contractility. TAPSE may increase by ~20% relative to baseline (~4–5 mm) and S′ by ~80% (~8–10 cm/s) [46]. Additionally, there is an expected increase in PA pressures, which may result from increased cardiac output in healthy subjects or elevated resistance in subjects with underlying cardiopulmonary disease. The ASE Guidelines set a cut-point for abnormal elevation in PA pressures as an increase ≥20 mmHg in estimated RVSP from baseline and/or a peak RVSP ≥ 50 mmHg [5].

The European Society of Cardiology and European Respiratory Society recognize that there is a dynamic cardiovascular response to exercise and suggest calculating MPAP/CO slope with a value > 3 mmHg/L/min, among other invasive findings [47]. There is no direct comparison of invasive and noninvasive assessment of MPAP/CO slope; however, the use of >3.5 mmHg/L/min on echocardiogram is suggested based on the reduced precision of Doppler measurements [48] and observations in a large prospective series of patients [49]. The TAPSE/PASP ratio is expected to decrease with exercise; however, it has not been shown to have a relevant prognostic implication beyond that of the resting ratio [49].

## 6. Alternative Noninvasive Imaging Modalities

Even though cMRI provides superior resolution in anatomic definition and tissue characterization of the right heart, its limitations in the assessment of right heart pathophysiology include high-cost, inaccessibility in some practice settings, a lack of portability, and the technical challenge of breath-holding in dyspneic patients. The use of gadolinium is also contraindicated in patients with significant renal dysfunction. While CT imaging can clearly define right heart anatomy, it is associated with high radiation exposure and similar to cMRI, the use of iodinated contrast is also contraindicated in patients with advanced renal disease. The disadvantages of utilizing nuclear techniques are radiopharmaceutical exposure, poor spatial resolution compared to echocardiography, and inability to evaluate RV–PA coupling. Finally, although PET imaging is associated with high temporal and spatial resolution, its accuracy is limited by motion artifacts and partial volume effects. Furthermore, it is costly when compared to echocardiography, is cyclotron-dependent, and is not widely available. Thus, echocardiography has the advantage over these imaging modalities when information per unit cost is considered.

## 7. Selected Right Ventricular Failure-Associated Conditions

### 7.1. Acute Right-Sided Heart Failure

Acute right-sided heart failure is characterized by acute RV dysfunction without chronic adaptations such as RVH or remodeling such as apical dilation. There are several patterns of findings on echocardiogram that suggest an acute etiology rather than a chronic process. Acute PE, RV infarction, and RV trauma are specifically discussed. Acute right heart failure will present with RV dilation without concurrent RA dilation or IVC dilation; in four chamber images, the RV/LV ratio may be >1.1 at the cardiac base. There may be evidence of RV overload without concurrent PH. Regional wall motion abnormalities, specifically hypokinesis or akinesis of the mid RV free wall, may be present with or without additional evidence of RV systolic dysfunction.

In acute PE, typical findings include RV/LV ratio > 1.1 at the cardiac base, a short PA acceleration time when the estimated RVSP < 60 mmHg, and akinesis of the mid-RV free wall, which is known by the eponym McConnell’s sign in this clinical scenario. As demonstrated in a prospective cohort of 100 consecutive subjects with a clinical suspicion of acute PE, these findings are relatively specific and carry a high positive predictive value in patients without prior cardiorespiratory disease [50]. The RV EI may be ≥1 during both ventricular systole and diastole, suggestive of RV pressure overload. Hemodynamically unstable patients are more likely to demonstrate at least one of these findings, though approximately 2/3 of patients with a documented pulmonary embolism have no significant abnormalities on echocardiogram to suggest PE [51,52]. In patients with previously diagnosed cardiopulmonary disease, these findings are all less predictive of the diagnosis of acute PE. In a post-hoc analysis of 422 subjects with intermediate or high-risk PE, evidence of RV–PA uncoupling (TAPSE/PASP ratio < 0.34) in the setting of intermediate or high-risk PE is associated with an increased risk of early clinical deterioration and 30-day mortality [53]. Furthermore, point-of-care ultrasound (POCUS) in patients with a PE has been shown to predict 30-day adverse outcomes if there was concomitant RV dilatation in a retrospective cohort of 161 patients [54]. Finally, a thrombus may be visualized in the RA, RV, in transit across the tricuspid or pulmonic valve, or MPA.

**Figure 5:**
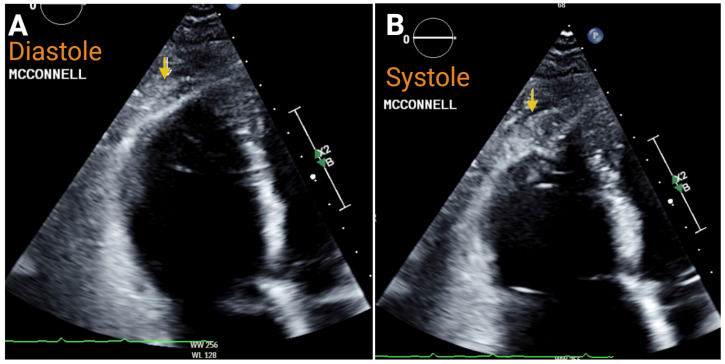
The 2DE diastolic Panel (**A**) and systolic Panel (**B**) views of the right ventricle, demonstrating akinesis of the mid free wall during acute pulmonary embolism. The yellow arrow indicates apical segment during diastole and systole, where there is normal systolic wall motion. Created in https://BioRender.com.

Many of the findings of an acute PE also hold true for RV infarction (Figure 5). It is worth recognizing that RV failure is often present in the setting of left ventricular infarction and is not specific for RV infarct [55].

**Figure 6:**
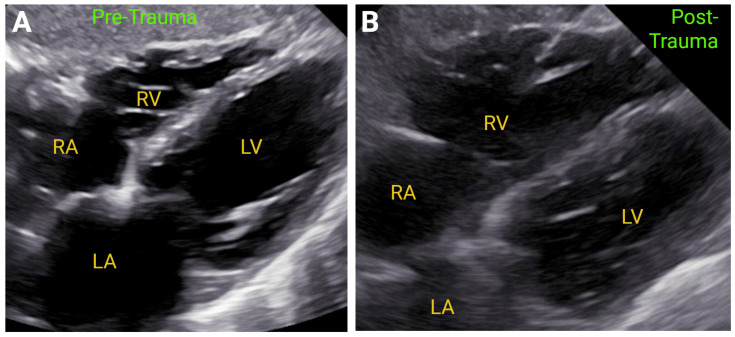
Representative 2DE views of the right ventricle, depicting normal right ventricular anatomy (RV) before chest trauma Panel (**A**) and RV dilation after chest compression-induced trauma to the sternum and mid RV free wall Panel (**B**). LA—left atrium. LV—left ventricle. Created in https://BioRender.com.

Chest trauma may cause similar findings to acute PE and RV infarction, including RV dilation and RV free wall hypokinesis in many instances (Figure 6). Significant TR can also be present due to a disrupted tricuspid annulus and torn tricuspid valve and apparatus. Furthermore, the echocardiogram should also assess for more foreboding features such as septal or free wall rupture, aortic rupture or dissection, and pericardial effusion and/or cardiac tamponade, potentially due to hemopericardium. Autopsy studies in deceased persons following severe blunt chest trauma found cardiac trauma in 15–20% of cases, with chamber rupture occurring in 36–65% of these cases. Another 15–17% of patients suffered injury to the great arteries, whereas valvular rupture is more sporadic [56]. It should be recognized that trauma to the chest may preclude TTE and that, if feasible, transesophageal echocardiography (TEE) may provide additional information [57].

**Figure 7:**
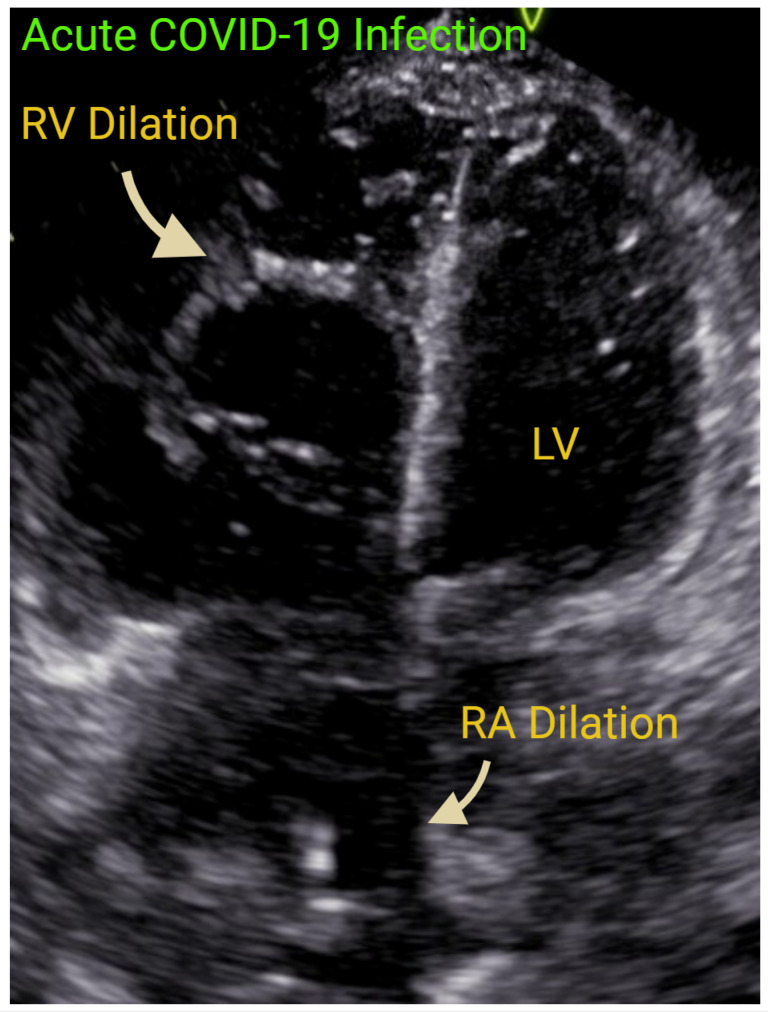
A 2DE view depicting significant right ventricular (RV) dilation during acute COVID-19 infection. Right atrial enlargement is also present. COVID-19—Coronavirus Disease 2019. RA—right atrium. LV—left ventricle. Created in https://BioRender.com.

The RV, more than the LV, has been demonstrated to suffer insults during acute Coronavirus-2 (COVID-19) infection [58] (Figure 7). An observational retrospective study of 90 mechanically ventilated subjects with COVID-19 infection showed that 49% to 70% of patients had RV dilatation and up to 40% had RV dysfunction [59]. Interestingly, COVID-19 patients were likely to develop radial RV dysfunction with more elevated PASP [60]. Perhaps this was observed because these patients had concomitant pulmonary and thrombo-occlusive disease. RV function in this population group was predictive of mortality. In a systematic review of 641 subjects from 7 studies, it was determined that every 1 mm reduction of TAPSE was associated with a 20% mortality increase [61]. Endorsed by ASE, POCUS was especially useful in the care of COVID-19 patients [62].

### 7.2. Chronic Right Ventricular Failure

Chronic right-side heart failure can result from chronic pressure, volume, or combined overload. The pathologic process is a continuum; thus, in the early phase, concentric RVH can be present and, in the late stage, the RV myocardium can be thinned. In chronic volume overload, the RV initially can absorb the increased preload without a significant rise in pressure, but, over time, the tricuspid annulus will dilate, leading to worsening tricuspid regurgitation and exacerbating the cycle. Notably, 2DE, 3DE, and TEE can show RV dilatation with increased EI and leftward septal shift. Hemodynamically, the severity of TR and RSVP initially are increased and then will gradually decrease to the “normal” range when RV systolic function becomes severely reduced. In an observational prospective cohort of 513 patients with secondary TR, the effective RV ejection fraction ((RV forward stroke volume–tricuspid regurgitant volume)/RV end-diastolic volume) has been shown to have better association with all-cause mortality and HF hospitalizations when compared to RV ejection fraction, TAPSE, RVFWS, and RVFAC [63].

#### 7.2.1. Pulmonary Hypertension

**Figure 8:**
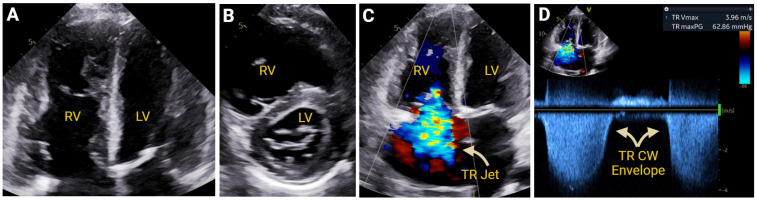
Representative 2DE view depicting severe right ventricular (RV) enlargement Panel (**A**) and flattening of the interventricular septum seen in RV pressure overload Panel (**B**). Color Doppler demonstrating severe tricuspid regurgitation (TR) Panel (**C**) and continuous wave (CW) Doppler measurement of severely elevated pulmonary artery systolic pressure Panel (**D**). Created in https://BioRender.com.

TAPSE, RVFAC, RVFWS, and TDI systolic velocities have been well-validated in PH screening and in PH prognostication [47]. In 290 pulmonary arterial hypertension patients entered into the Giessen Pulmonary Hypertension Registry, the TAPSE/PASP ratio can be used to stratify hemodynamics categories (low < 0.19 mm/mmHg; moderate 0.19–0.32 mm/mmHg; high > 0.32 mm/mmHg), with patients in the low tertile predicted to have increased morbidity and mortality [64] (Figure 8).

#### 7.2.2. Chemotherapy

TAPSE and RVFAC have been shown to decrease prior to RA size and LV function changes in patients receiving anthracycline chemotherapy for breast cancer [65]. In a small study (n = 28) of low-cardiovascular-risk patients treated with anthracycline, changes in RVFWS were evident prior to LV global longitudinal changes were detected [66]. In a meta-analysis of 25 studies (n = 1520), RV function, RV GLS and FWS, and TAPSE significantly decreased while PASP significantly increased in patients exposed to cardiotoxic chemotherapeutic agents [67]. Thus, the assessment of RV function may provide an early signal on the impact of therapy before LV systolic function develops, which can then permit earlier intervention.

#### 7.2.3. Simple Congenital Heart Defects

**Figure 9:**
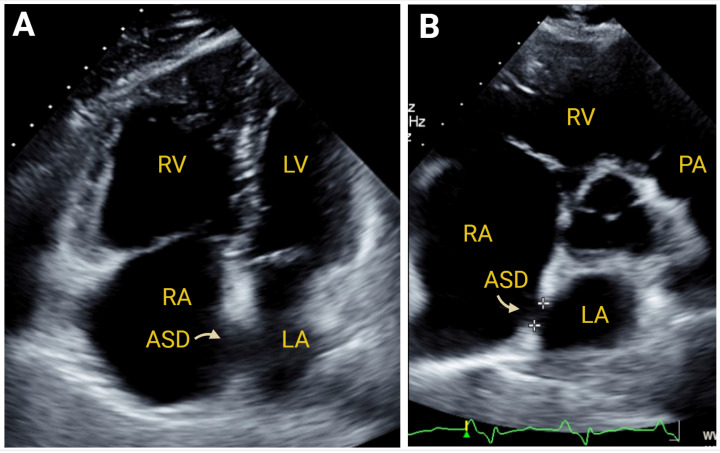
A 2DE image of right ventricular (RV) enlargement in the presence of a secundum atrial septal defect (ASD) in the apical 4-chamber view Panel (**A**) and in the parasternal short-axis view Panel (**B**). RA—right atrium. LA—left atrium. LV—left ventricle. PA—pulmonary artery. Created in https://BioRender.com.

**Figure 10:**
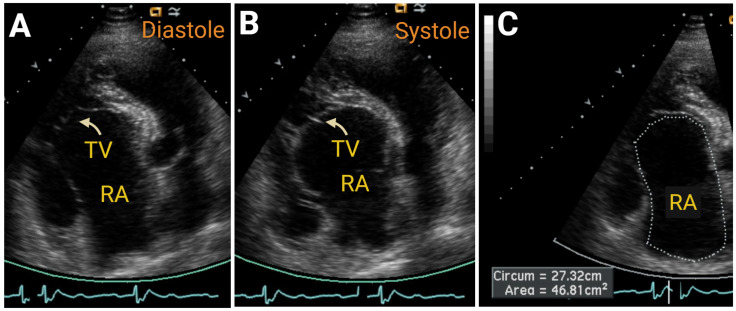
A 2DE image of Ebstein’s anomaly in diastole Panel (**A**) and in systole Panel (**B**), depicting severely dilated right atrial (RA) size Panel (**C**). TV—tricuspid valve. Created in https://BioRender.com.

Chronic right heart failure can also be resultant from congenital heart defects. It is beyond the scope of this review to provide a comprehensive discussion, but, of note, hemodynamically significant atrial septal defects [68] (Figure 9), Ebstein’s anomaly [69] (Figure 10), and patent ductus arteriosus [70] are simple congenital lesions that can be readily diagnosed with 2DE, 3DE, and Doppler imaging.

#### 7.2.4. Pulmonic and Tricuspid Valvular Disease

Both primary and secondary pulmonic and tricuspid valve disease can lead to chronic right heart failure. Of note, 2DE, 3DE, and TEE can elucidate the etiology by demonstrating stenotic (congenital, rheumatic, and carcinoid) and regurgitant (myxomatous degeneration, prolapse, carcinoid, infectious endocarditis, traumatic rupture, and device lead impingement) lesions. As the RV experiences chronic pressure and volume overload, RV–PA uncoupling will develop and eventually lead to right heart failure. Echocardiography is the first-line diagnostic tool in these individuals that will permit a determination of not only the etiology but also the sequelae of untreated pathology [4,71,72].

### 7.3. Cardiomyopathies

There are some cardiomyopathic processes which fall outside the standard adaptations seen with RV failure due to left-sided HF or primary PH. These include HCM, cardiac amyloidosis, and ARVC. Right-sided involvement in HCM is increasingly recognized and clinically portentous. Findings consistent with HCM include RVH, a reduction in RV strain on STE, mildly reduced TAPSE, and potential RV–PA uncoupling. Abnormal strain may be present in about one quarter of patients with HCM, while more traditional measures of RV dysfunction are present in 5–10% of cases [73,74]. Patients with HCM may benefit from a treadmill exercise assessment of RV parameters, particularly assessing RV strain values, which are markedly abnormal compared to healthy controls. In two small cohorts of 45 HCM patients and 28 HCM patients with RV hypertrophy, abnormal augmentation in RV contractility during exercise in patients with HCM suggests impairment in RV contractile reserve and correlates with reduced exercise capacity [75,76].

**Figure 11:**
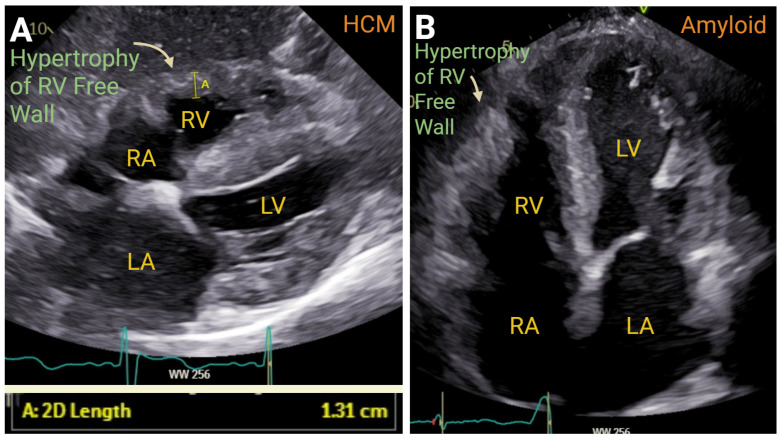
A 2DE image of right ventricular hypertrophy (RVH) in hypertrophic cardiomyopathy (HCM) Panel (**A**) and in cardiac amyloidosis Panel (**B**). RA—right atrium. RV—right ventricle. LA—left atrium. LV—left ventricle. Created in https://BioRender.com.

Like HCM, cardiac amyloid may be detected by increased RVWT without concurrent PH. Cardiac amyloid may also demonstrate only mild RV systolic dysfunction, with more prominent abnormalities in RVFWS being common. Notably, RVFWS is most impacted at the base with relative apical sparing, a finding which has also been described in the LV. This apical sparing feature of RVFWS may help to distinguish cardiac amyloid from HCM on echocardiogram [77,78]. Despite modest RV dysfunction and PH, an abnormal TAPSE/PASP ratio predicted HF hospitalization and mortality in one Italian series of 283 patients with cardiac amyloid [79] (Figure 11).

Echocardiographic findings in ARVC include RV dilation, decreased RV systolic function, and morphologic abnormalities such as a hyper-reflective moderator band. In contrast to both HCM and cardiac amyloid, ARVC is not characterized by RVH; in fact, ARVC is more likely to mimic the findings of a left to right shunt (chamber dilation without hypertrophy). The diagnosis of ARVC was redefined in 2010 with the Padua criteria. To meet the Padua major echocardiographic criteria, a patient must demonstrate regional RV akinesia, dyskinesia, or aneurysm and either a FAC ≤ 34% or RV dilation at the RVOT (≥32 mm in PLAX or ≥36 mm in PSAX views) [80]. Beyond these specific criteria, RV strain may predict progressive deformation and an increased risk of arrhythmias [81].

## 8. Conclusions

Despite the advances in cMRI, echocardiography remains the first-line imaging modality for the diagnosis of acute and chronic right heart failure. Virtually all 2D and Doppler parameters have well-established prognostic significance and are relevant diagnostic tools in the determination of the etiology of RV dysfunction, screening for pulmonary hypertension, and assessment of disease regression and progression and response to therapeutic interventions in right heart failure. In particular, 3D echocardiography is relied upon to provide an accurate assessment of RV ejection fraction. Conventional echocardiography and POCUS are versatile and expedient imaging tools that can readily be utilized in the outpatient setting, the emergency department, and the hospital setting, including the critical care units and operating rooms, for the assessment of right heart anatomy and function. Integration of traditional hemodynamic measurements with advanced ultrasound modalities, particularly STE with RA and RV strain analysis, have advanced the ability to determine the pathophysiology at the bedside and have positively impacted clinical care by providing immediate data to providers to guide treatment decisions. A contemporary echocardiogram in a patient with right heart failure must include 2D and 3D measurements of the right chambers size and wall thickness; determination of RV systolic function using 2D-derived FAC, 3D-derived RV ejection fraction, and RV free wall GLS; estimation of RAP using IVC and/or hepatic vein interrogation; calculation of RVSP, mPAP, and PAEDP if possible; and the assessment of the tricuspid and pulmonic valve function and any abnormal anatomy that could be contributing to the failing right heart.

## 9. Future Directions

Artificial intelligence (AI) promises to revolutionize echocardiography image acquisition and interpretation. Efforts are underway to apply AI to nearly every aspect of echocardiography, from the assessment of 2DE conventional measurements and 3DE volumetric analysis to mining data for variables indicative of early disease states [82]. When AI-derived measurements of FAC, FWS, and TAPSE were compared with cMRI (RVEF < 40%) to evaluate RV dysfunction in 225 patients, it had excellent sensitivity (91%) and negative predictive value (96%) but lower specificity (50%) and positivity predictive value (32%). Thus, AI could assist in rapid screening in acute settings to exclude RV dysfunction [83]. In addition to surrogate markers of RV function, the AI-derived analysis of RV volumes from 3DE had a high sensitivity of 94% in the identification of reduced RVEF with a cut-off value of 43% when compared with cMRI [84]. Global, longitudinal, and septal AI-assisted strain analysis also correlated well with TAPSE, FAC, and tissue Doppler S′ in assessment of RV function [85]. Furthermore, the AI-generated assessment of RV–PA coupling parameters has been shown to correlate positively with invasive right heart catheterization measurements in defining the prognostic risk factors in a large cohort of 737 patients with severe tricuspid regurgitation undergoing tricuspid valve interventions [86]. Accordingly, the AI-assisted assessment of the right heart has great potential to improve diagnostic and prognostic accuracy by reducing intra- and inter-observer variability and to facilitate efficient care delivery. However, ongoing validation studies are needed to further refine accuracy in various population groups and to quantify hemodynamic load in disease states at different timepoints. Finally, AI-guided and protocol-driven echocardiography can aid image acquisition, which can compensate for variable operator skillsets and improve image quality to produce high-yield diagnostic images [87].

The views expressed in this presentation reflect the results of research conducted by the author(s) and do not necessarily reflect the official policy or position of the Department of Veterans Affairs or the U.S. Government.

## Figures and Tables

**Table 1:** Correlation between echocardiographic measurements and cardiac MRI.

Measurement	ASE Reference Values				Correlation with MRI	ROC Curve
	Abnormality Grading					
	Normal	Mild	Moderate	Severe		
**Right Atrial Size**						
Area, cm^2^	<19	19 to 22	23 to 24	>24		
Area to length method, mL/m^2^	<30	30 to 36	37 to 41	>41		
Method of disks, mL/m^2^	<33	33 to 38	39 to 44	>44	r = 0.96	
**Right Ventricular Hypertrophy**						
RVWT (subcostal), cm	<0.5	0.5 to 0.7	0.8 to 0.9	>0.9	r = 0.71	
**Right Ventricular Size**						
RV basal diameter (A4C), cm	<41	41 to 44	45 to 49	>49	r = 0.69 to 0.73	AUC = 0.92
Proximal RVOT (PSAX), cm	<34	34 to 38	39 to 41	>41	r = 0.68	
RV end to diastolic area (A4C), cm^2^	<25	25 to 28	29 to 32	>32	r = 0.76	AUC = 0.94
**Right Ventricular Systolic Function**						
TAPSE, cm	>1.7	≤1.7 to ≥1.3	≤1.3 to >1.0	≤1.0	r = 0.45 to 0.62	AUC = 0.74
S′, cm/s	>9.5	≤9.5 to ≥7.2	≤7.2 to >5.0	≤5	r = 0.36 to 0.52	
FAC, %	>35	≤35 to >29	≤29 to >22	≤22	r = 0.55 to 0.77	AUC = 0.83
RV FWS, %	>20	≤20 to <15	<15 to ≥11	<11	r = 0.69 to 0.92	
3D RVEF, %	>45	≤45 to <39	≤39 to ≤32	<32	r = 0.56 to 0.95	

ASE—American Society of Echocardiography; MRI—magnetic resonance imaging; ROC—receiver operating characteristic; RVWT—right ventricular wall thickness; RV—right ventricular; A4C—apical 4-chamber; RVOT—right ventricular outflow tract; PSAX—parasternal short axis; TAPSE—tricuspid annular plane systolic excursion; S′—tissue Doppler peak systolic velocity; FAC—fractional change area; 3D RVEF—three-dimensional right ventricular ejection fraction.

## Data Availability

No data sets were generated or analyzed during the current study.
